# Double Cystic Artery Originating From the Superior Mesenteric Artery and Right Hepatic Artery: A Case Report

**DOI:** 10.7759/cureus.76536

**Published:** 2024-12-28

**Authors:** Tanish Rao, Saransh K Saini, Chamath Ranaweera, Lakal Dissabandara

**Affiliations:** 1 Medicine, Northern Hospital Epping, Melbourne, AUS; 2 Medicine, Gold Coast University Hospital, Gold Coast, AUS; 3 Surgery, Sunshine Coast Hospital and Health Service, Sunshine Coast, AUS; 4 Medicine, Griffith University, Gold Coast, AUS

**Keywords:** anatomical variation, cadaver, calot's triangle, cholecystectomy, cystic artery, dissection

## Abstract

The cystic artery is a critical anatomical landmark in both laparoscopic and open cholecystectomy. This report presents a unique case involving two rare anatomical variations: double cystic arteries, along with a superficial branch originating from the superior mesenteric artery (SMA) - a previously unreported combination with significant clinical and surgical implications. Unlike earlier studies, this research provides detailed anatomical and embryological insights supported by high-quality imaging and illustrations to guide surgeons in recognizing and managing this novel variation. The cadaver examined in this study was donated to the Griffith University School of Medicine for medical education and research. A macroscopic examination was conducted to identify anatomical variations and elucidate the relationships between the atypical cystic artery and surrounding abdominal structures. The typical cystic artery originated from the right hepatic artery, passing posterior to the common hepatic duct within the hepatocystic triangle to supply the superomedial (dorsal/deep) surface of the gallbladder. An accessory cystic artery (100 mm long, 2.5 mm in diameter) originated 35 mm distal to the SMA origin, with a retro-pancreatic and sub-hepatocystic course, bifurcating to supply the cystic duct and the inferolateral (superficial) surface of the gallbladder. This configuration, resembling an aberrant right hepatic artery in size and position, poses heightened risks of bleeding and injury during laparoscopic hepatoduodenal ligament dissection, duodenal mobilization, and in the presence of pancreatic inflammatory or neoplastic processes. By addressing a significant gap in the literature, this study advances both anatomical knowledge and surgical safety. Understanding such variations can significantly improve the safety and efficacy of cholecystectomies and other hepatobiliary, duodenal, and pancreatic surgical procedures. In select cases, preoperative imaging such as computed tomography angiography and collaboration with radiologists can aid in detecting vascular anomalies and guiding surgical planning.

## Introduction

This article was previously posted to the Research Square preprint server on 30 May 2024 [1].

Given that laparoscopic cholecystectomy is the gold-standard treatment option for symptomatic gallstone disease [2], surgeons must have an extensive understanding of hepatobiliary vascular variations, with the cystic artery being the second most variable branch in the extrahepatic vasculature after the right hepatic artery (RHA) [3]. Conversion to open cholecystectomy due to vessel injuries occurs in 1.9-6.2% of laparoscopic cases, with a mortality rate of approximately 0.02% [2,4]. Cystic artery injuries alone account for 1.5 conversions to open surgery per 1,000 procedures [3]. Laparoscopic repair of vessel injuries is feasible and safe for experienced surgeons if the bleed is small, easily localized, and addressed before excessive blood obscures the operative field [2]. For difficult cases, a subtotal cholecystectomy may reduce the risk of vascular or biliary injury, though it increases the risk of postoperative bile leaks, abdominal fluid collections, and reoperations [5].

The cystic artery usually arises from the RHA, passing anterior to the portal vein and posterior to the common hepatic duct (CHD) within the hepatocystic triangle, where it lies anterosuperior to the cystic duct (CD), often splitting into a superficial and deep branch [6]. The deep branch typically runs between the gallbladder and gallbladder fossa, while the superficial branch supplies the opposing surface along the gallbladder’s left side [3].

However, several well-documented variations exist in the cystic artery’s origin, course, and position exist [3-6]. A comprehensive 2016 review by Andall et al. [3] examining over 9800 cases through cadaveric dissection, CT angiography, and intraoperative studies found that only 79.02% of cystic arteries originate from the RHA and 81.5% pass through the hepatocystic triangle. In 8.9% of cases, multiple cystic arteries were observed, and in 4.9%, the cystic artery ran inferior to the CD/hepatocystic triangle. Other origins for the cystic artery included the left hepatic artery (LHA), aberrant RHA, gastroduodenal artery (GDA), proper hepatic artery (PHA), coeliac trunk (CT), proper hepatic bifurcation, superior pancreaticoduodenal, and the superior mesenteric artery (SMA). A cystic artery originating from the SMA is an exceptionally rare anomaly, with only 20 cases (0.29%) reported in the study, all involving a single/replaced cystic artery. Two recent systematic reviews identified an additional 17 cases of single/replaced cystic arteries originating from the SMA, estimating a slightly higher prevalence of 1.09-1.25% [4,5]. However, these reviews provided little additional information beyond the rarity of this variation, attributing this to insufficient reporting of cystic artery anatomy, including its origin, morphology, relationship to the hepatocystic triangle and the biliary tree, length, and diameter [5].

The remarkable variability observed in hepatobiliary vasculature arises from its complex embryological development. The hepatic and cystic diverticula form during the third gestational week. Although considered foregut-derived structures, they are located at the foregut-midgut junction during development, commonly receiving arterial supply from the 10th to 13th vitelline segments [4]. Between the fourth and seventh gestational weeks, the vitelline arteries arising from the fused dorsal aorta are connected ventrally by Tandler's longitudinal anastomosis, where the 10th and 13th segments give rise to the CT and SMA, respectively [7]. The branching patterns of the CT and SMA depend on the obliteration pattern of the ventral anastomosis, as well as the variable growth and rotation of the liver, pancreas, stomach, and duodenum. Initially, the primitive liver is supplied by an aberrant LHA from the left gastric artery (LGA), an aberrant RHA from the SMA, and a middle hepatic artery - the future common hepatic artery (CHA) - without branches. Obliteration of the 11th and 12th vitelline segments typically leads to the regression of the aberrant hepatic arteries, while the middle hepatic artery branches into the LHA and RHA, forming the CHA and PHA [8,9]. Variations in the persistence and regression of portions of the ventral anastomosis are hypothesized to cause the diverse branching patterns observed in hepatic and cystic arterial supply [4,6-9].

In vascular anatomy, an "accessory cystic artery" refers to an anomalous artery supplementing the regular cystic artery's blood supply, whereas a "replaced cystic artery" denotes a similar anomalous artery exclusively nourishing the gallbladder. A double cystic artery refers to cases in which the usual superficial and deep branches of the cystic artery arise from separate origins [3,6,10]. So, although commonly labeled as branches, they represent distinct arteries. In double cystic artery cases, the deep branch usually arises from the RHA, with the variation generally being in the superficial branch [3,6,10]. The superficial branch has only been reported to arise from CT tributaries (i.e., RHA, LHA, CHA, GDA, and retroduodenal artery) [11], but never from the SMA.

This case study is the first to document a double cystic artery with a superficial branch arising from the SMA. Unlike previous reports, which focus on single/replaced cystic arteries with limited anatomical detail, this study aims to provide comprehensive descriptions of the origin, morphology, relationships to the hepatocystic triangle and the biliary tree, length, and diameter of this novel variation. By exploring the embryological basis of this variation and its clinical implications, this study aims to enhance the understanding of cystic artery anomalies and their surgical relevance, particularly for cholecystectomies and other hepatobiliary, duodenal, and pancreatic procedures.

## Case presentation

The cadaver was that of a 91-year-old Caucasian male. Notable evidence of disease during dissection included the presence of multiple jejunal diverticula, bowel adhesions, and a peritoneal dialysis port. During a student elective dissection project at the Griffith University School of Medicine, we observed an abnormal branching pattern of the cystic artery. The branching patterns of the CT, SMA, and extrahepatic biliary tree were carefully examined. The cystic artery's branching pattern and its positional relationships to the gallbladder, intestines, biliary tree, and vasculature were studied in detail. The cadaver exhibited no signs of intestinal malrotation.

**Figure 1:**
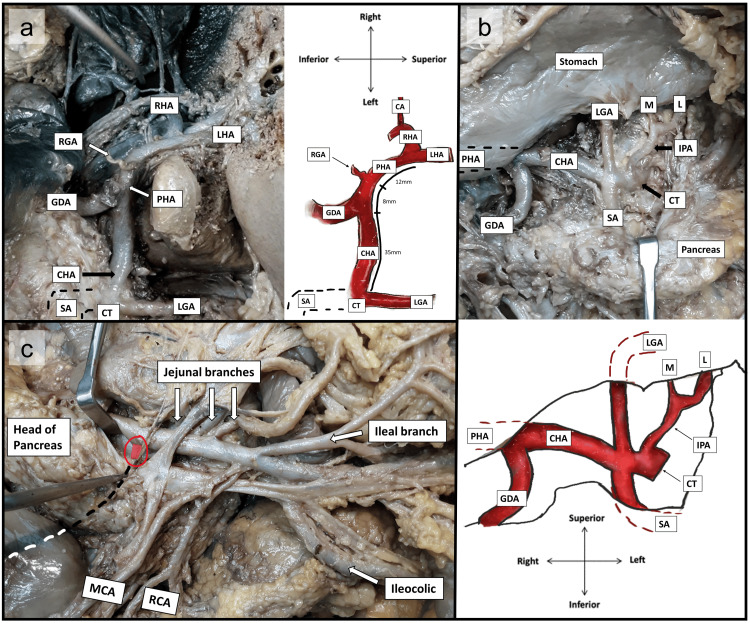
Prosection of the coeliac trunk and super mesenteric artery along with all anatomical relations. (A-B) Prosection and scaled anatomical illustration depicting the four branches of the coeliac trunk and the branching pattern of the CHA, respectively. (C) Prosection of the SMA and its branches, with the SMA and pancreatic head separated to expose the aCA origin. SMA: superior mesenteric artery; aCA: accessory cystic artery; CT: coeliac trunk; IPA: inferior phrenic artery; M: medial branch of IPA; L: lateral branch of IPA; LGA: left gastric artery; CHA: common hepatic artery; PHA: proper hepatic artery; GDA: gastroduodenal artery; SA: splenic artery; RGA: right gastric artery; LHA: left hepatic artery; RHA: right hepatic artery; red circle: origin of aCA off the SMA; pink: accessory cystic artery (visible portion); dotted line: path of accessory cystic artery; RCA: right colic artery; MCA: middle colic artery.

**Figure 2:**
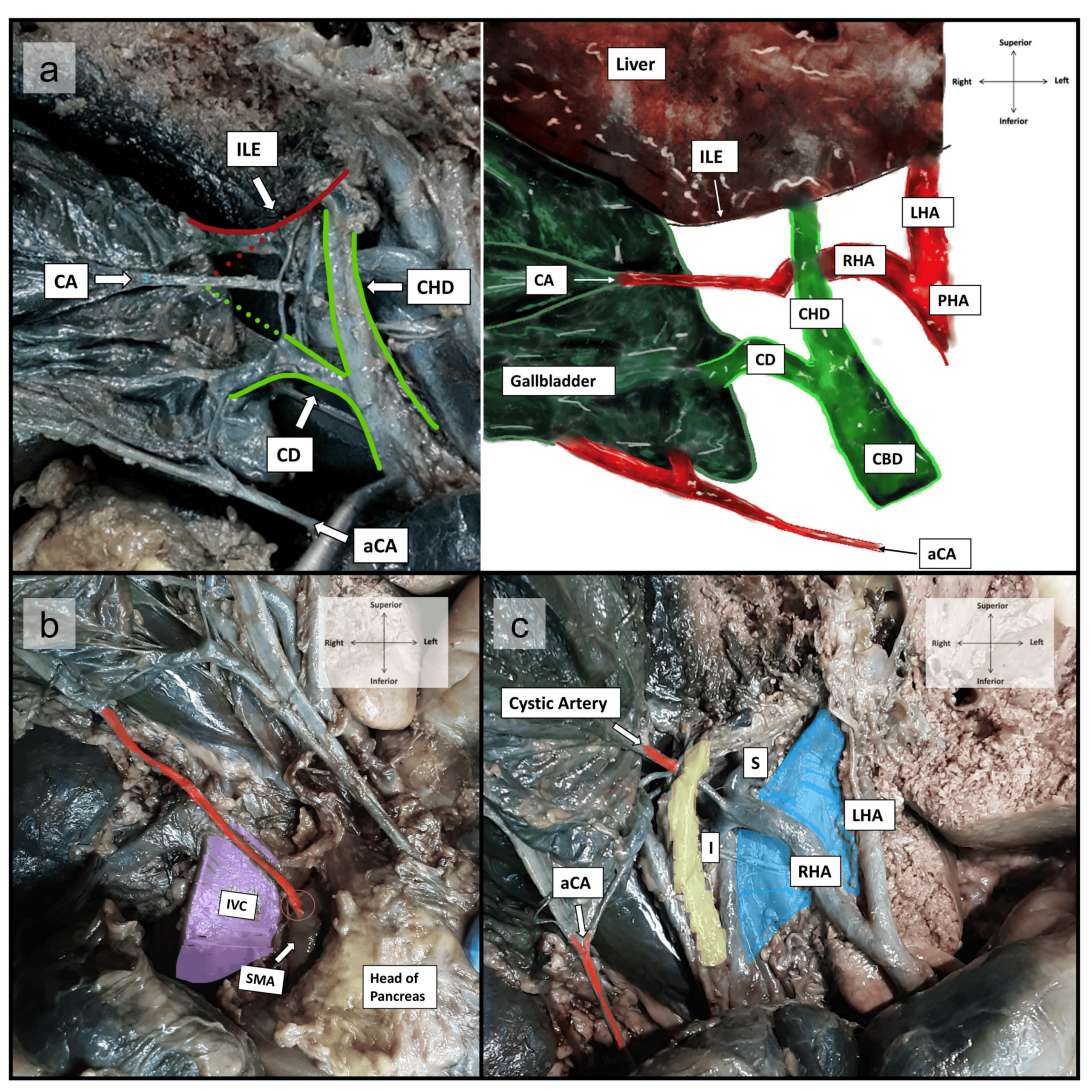
Prosection of the normal and accessory cystic artery along with all anatomical relations. (A) Prosection and scaled anatomical illustration of Calot’s triangle with both the normal and accessory cystic arteries and their anatomical distribution. Note the position of the aCA outside/below Calot’s triangle, compared to the normal CA located within the triangle. The borders of this triangle include the ILE superiorly, the CHD medially, and the CD inferiorly. (B) Prosection of the entire course of the aCA originating from the SMA, with the duodenum and pancreatic head reflected inferiorly to aid visualization of the aCA origin and course. (C) Dissection of the hepatoduodenal ligament to expose the portal triad and its anatomical distribution. PHA: proper hepatic artery; LHA: left hepatic artery; RHA: right hepatic artery; light blue: portal vein; IVC: inferior vena cava; purple: IVC; S: superior branch of RHA; I: inferior branch of RHA; yellow: common hepatic duct; aCA: accessory cystic artery; CA: cystic artery; ILE: inferior liver edge; CHD: common hepatic duct; CD: cystic duct; CBD: common bile duct; orange circle: origin of aCA off the SMA; red: accessory cystic artery; SMA: superior mesenteric artery.

Figure 1B depicts four branches of the CT. Most proximally is the left inferior phrenic artery (IPA), which bifurcates into medial and lateral branches. Distally, the CT trifurcates into the LGA superiorly, the CHA to the right, and the splenic artery (SA) inferiorly. Approximately 35 mm from its origin, the CHA gives off a gastroduodenal branch. It continues as the PHA for approximately 8 mm before giving off a right gastric artery (RGA) branch (transected in Figure 1A) and another 12 mm before bifurcating into the LHA and RHA. As depicted in Figure 2C, both hepatic arteries lie ventral to the portal vein. The LHA runs along its medial surface, while the RHA traverses horizontally across it and dorsal to the CHD. The RHA subsequently trifurcates into a superior, inferior, and cystic artery branch, supplying the gallbladder's superomedial body (dorsal surface). Figure 2A below clearly demonstrates the location of this cystic artery within the hepatocystic triangle, defined by the inferior liver edge (ILE) superiorly, the CHD medially, and the CD inferiorly.

The accessory cystic artery (aCA), an anatomical variant, lies outside the triangle and inferior to the common hepatic duct and CD. It bifurcates before reaching the gallbladder, with one branch supplying the CD and another traversing the inferolateral gallbladder neck. Figure 2B highlights the aCA (marked in red) originating from the posteromedial aspect of the SMA. Measuring approximately 100 mm in length, the artery traverses the dorsal aspect of the pancreatic head and, upon exiting, courses posteroinferior to all structures of the portal triad. Figure 2B shows the aCA dissected, with the pancreatic head retracted to improve visualization of this structure. Conversely, in Figure 1C, the pancreas is shifted laterally to better visualize the remaining SMA branches: the jejunal, ileal, ileocolic, right colic artery (RCA), and middle colic artery (MCA) branches in a clockwise order. The origin point of the aCA (highlighted in pink) from the SMA is visualized and circled in red. A dotted line traces the path of the aCA, originating approximately 35 mm distal to the SMA origin and 10 mm proximal to the first jejunal artery. The aCA was slightly thicker (2.5 mm at its origin and 2 mm at its termination before dividing) compared to its standard counterpart (1.5 mm throughout); however, it did not display any evidence of asymmetric thickening, stenosis, or vascular endothelial swelling. Given that each cystic artery supplied opposing surfaces of the gallbladder, this cadaver exhibited a double cystic artery, with the aCA homologous to the superficial branch and the normal cystic artery homologous to the deep branch.

## Discussion

Given the low incidence of cystic arteries arising from the SMA, it is important to carefully evaluate all reported cases of this variation in the literature, including both cross-sectional studies and case reports. Notably, these reports consistently describe a single replaced cystic artery, unlike the accessory artery observed in our case. Among the 13 cross-sectional studies reporting this variation across three major systematic reviews [3-5], only a few provided anatomical details beyond prevalence statistics, as discussed below.

The seminal cadaveric study by Daseler et al. [12] identified a single instance of this variation among 500 cadavers. They described a “long” and “slender” vessel originating from the first portion of the SMA, ascending posterior to the duodenum, pancreas, portal vein, and common bile duct (CBD), before passing beneath the CD to insert at the gallbladder neck. Although resembling our case, their cystic artery traversed dorsal to the pancreatic head, whereas ours passed through it and remained beneath the CD. A preoperative CT-based study identified two cases among 78 patients [13], describing “long” vessels positioned shallowly, running inferior to the CD and outside the hepatocystic triangle, findings consistent with the sub-hepatocystic positioning of our aCA. Similarly, another study reported one case among 72 cadavers [14] noting that the artery lay outside the hepatocystic triangle. In a cadaveric study of 50 specimens [15], a single case described the artery passing through the pancreatic head, aligning with our findings. In contrast, another study involving 30 cadavers [16] described a 24.17 mm artery positioned anterior to the CHD and within the hepatocystic triangle, differing significantly from our case, where the aCA was both longer and situated lower.

Individual case studies further illustrate this variability. One describes a patient with variant CT anatomy and a 130 mm long cystic artery that ran dorsal to the portal vein, passed through the hepatocystic triangle, and supplied the dorsal surface of the gallbladder [17]. Another case describes a 35 mm long replaced cystic artery traveling dorsal to the portal vein and the CBD, entering the lower border of the hepatocystic triangle, and bifurcating into branches supplying the gallbladder body and CD [18]. Shared features with our case include a dorsal path crossing the portal vein and CBD, along with a low-lying position of the cystic artery below the hepatocystic triangle, though ours travels particularly low. As with the cross-sectional studies, variability is observed in the regions of the gallbladder supplied (ours passes through the pancreatic head rather than dorsal to it), the size of the cystic artery (e.g., as short as 35 mm in one case), and its position relative to the pancreatic head (ours traverses within it rather than dorsal to it). In our case, the more inferior path of the aCA to the gallbladder, the CD, and the hepatocystic triangle likely represents a double cystic artery anomaly to supply the superficial surface of the gallbladder, as the standard cystic artery already supplies the dorsal surface of the gallbladder. A notable case of cadaveric multi-organ procurement also described a replaced cystic artery arising from the SMA mimicked a replaced or accessory hepatic artery both clinically and pathologically, with a palpable pulse on the right side of the portal vein and a similar course and caliber to the replaced or accessory right hepatic arteries [19]. Additionally, patients with a replaced or accessory RHA arising from the SMA often exhibit multiple small branches supplying the gallbladder, unlike the single cystic artery observed in our case [20].

**Figure 3:**
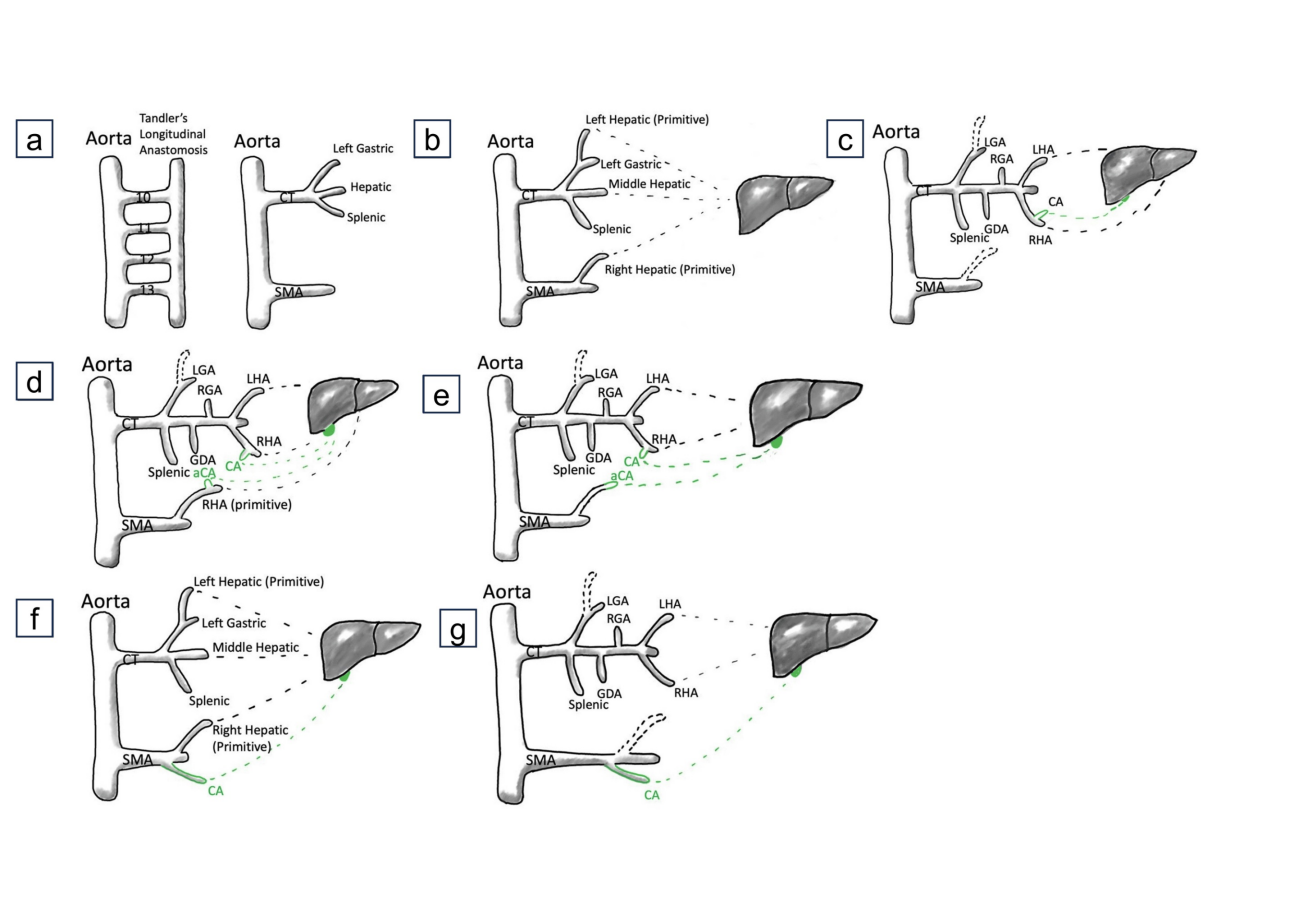
Embryological basis of the cystic artery variation. (a) Describes the vitelline arteries from the fused dorsal aorta joining ventrally by an anastomotic channel. Note the subsequent regression of the 11th and 12th vitelline segments, whereas 10 and 13 form the CT (with left gastric, hepatic, and splenic branches) and SMA, respectively [4,6-8]. (b) Describes the subsequent branches that supply the liver. A primitive left hepatic is off the left gastric, the middle hepatic is off the CT, and the primitive right hepatic is off the SMA [9]. (c) Describes the regression of the primitive right and left hepatic branches (dotted lines). The middle hepatic subsequently branches out into the left and right hepatic arteries, which now supply the liver. The diagram shows that the cystic artery commonly branches off the right hepatic. (d) Describes the likely embryological variation in our case. The primitive right hepatic also has a cystic artery branch (aCA), resulting in dual supply to the gallbladder. (e) While the primitive right hepatic branches to the liver vanish, the aCA remains, leading to a direct connection from the SMA to the liver. (f) Demonstrates an alternative case where the CA comes directly off the SMA (rather than the primitive right hepatic artery). Note that this is an aberrant artery, replacing the CA branch usually coming off the right hepatic. (g) The main distinguishing factor in this variation is that only one artery supplies the gallbladder. It is otherwise remarkably similar to our specimen. CT: coeliac trunk; SMA: superior mesenteric artery; aCA: accessory cystic artery; CA: cystic artery; SMA: superior mesenteric artery; LGA: left gastric artery; GDA: gastroduodenal artery; RGA: right gastric artery; LHA: left hepatic artery; RHA: right hepatic artery; RGA: right gastric artery. Image credit: Saini SK and Rao T.

Describing the embryological basis of cystic artery variations is complex, as illustrated in Figure 3 [4,6-9], especially given that our case displayed features of both typical and atypical arterial anatomy. Although our patient retained a normal cystic artery with standard common hepatic and coeliac trunk branching, the morphological similarity of our accessory vessels’ course to that of an accessory/replaced RHA suggests that the embryological basis of our aCA is linked to the persistence of a cystic artery from an aberrant RHA derived from the SMA in utero. Variations in the regression of the vitelline artery or ventral anastomosis would typically result in a replaced rather than an accessory cystic artery [10].

The modern laparoscopic cholecystectomy technique emphasizes establishing the critical view of safety to ensure precise identification of the cystic duct and artery before ligation. This view is achieved through gallbladder retraction and careful dissection of the hepato-duodenal ligament overlying the hepatocystic triangle, using established anatomical landmarks to define “safe” dissection zones. The view is complete when all fibro-fatty tissue is cleared from the hepatocystic triangle, the lower third of the cystic plate is exposed, and only two structures (the CD and cystic artery) are seen entering the gallbladder [21]. Currently, no universally accepted surgical classification system exists for cystic artery types. Most classifications focus on the number of cystic arteries and whether they are located within or outside the hepatocystic triangle during dissection [13,22]. However, these classifications often lack practical value for surgeons, as cystic arteries coursing outside the hepatocystic triangle can exhibit significant variability. While several common patterns in these variations are linked to the vascular origin of the aberrant or accessory cystic arteries, individual cases, as exemplified in the existing reports of single aberrant cystic arteries originating from the SMA [12-18], frequently show variability in their course. Our unique case of a double cystic artery further highlights the challenges these variations pose for unsuspecting surgeons.

A major surgical risk in our case is overlooking both cystic arteries, especially the fragile deep branch, which is prone to accidental transection during peritoneal dissection [20]. This branch is a common source of postoperative reactionary bleeding. Low-lying cystic arteries, such as the aCA observed in our case, are often the first structures encountered in laparoscopic procedures. Unlike their low-lying position in open procedures, they may appear superficial and anterior to the CD due to carbon dioxide (CO2) insufflation and retraction methods, making them more susceptible to injury during the initial hepatoduodenal ligament dissection, especially since they will be lying within the safe zone of dissection [23]. Surgeons unaware of this variation may mistakenly identify the cystic artery as the CD, resulting in bleeding and bile duct injury. Furthermore, in the anterior laparoscopic position, surgeons may frequently find it necessary to ligate vessels before visualizing the CD [3]. Typical cystic arteries also yield branches that extend to the CD, requiring vessel division to acquire an appropriate length of the CD for division. An unaware surgeon may overlook the branches provided by our aCA inferior to the hepatocystic triangle, potentially resulting in unexpected bleeding [23]. Laparoscopically, a single large cystic artery also requires more careful dissection due to the difficulty distinguishing it from an aberrant hepatic artery [19], with damage leading to potential liver ischemia and necrosis [2].

While CT angiography (CTA) is a functional, non-invasive tool for identifying cystic artery anomalies, it has been unable to provide accurate and reliable cystic artery visualization in 8% of cases [22]. Although this detection rate is reasonably high, it limits the feasibility of preoperative CTA as a routine practice without specific alternative indications. One such alternative indication is for preoperative imaging for duodenal or pancreatic procedures, where the vessels originating from the SMA may have more significant implications for unaware surgeons. Surgeons will encounter this vessel during kocherization of the duodenum and mobilization of the pancreas causing potential bleeding, though the risk of ischemic injury to the gallbladder is theoretically low unless the deep branch fails to produce sufficient collateral supply. In the case of pancreaticoduodenectomy (PD) procedures, the ischemic risk is even less of a concern as this procedure routinely requires a cholecystectomy [24]. Another noteworthy consideration is the involvement of the retro-pancreatic portion of the aCA in inflammatory or neoplastic processes of the pancreatic head, leading to aneurysmal dilatation and retroperitoneal bleeding. Accessing this retro-pancreatic portion for embolization can be challenging for interventional radiologists [24]. When assessing the utility of contrast-enhanced CT in PD procedures for detecting the more common aberrant RHA arising from the SMA, it has demonstrated a 98% detection rate - provided both the surgeon and radiologist actively search for it [25]. However, studies reveal that nearly half of these cases are identified only intraoperatively due to inconsistencies in radiology reporting and surgeons' review of the imaging [25,26], a challenge we anticipate would similarly apply to our case. Hence, surgeons should not rely solely on imaging but also maintain a high index of suspicion intraoperatively. Close collaboration with radiologists and a thorough review of imaging studies are recommended to enhance the detection of such variations.

## Conclusions

This study documents the first known case of a double cystic artery with the superficial branch arising from the SMA. Unlike earlier reports, it offers a comprehensive analysis, including detailed anatomical descriptions, high-quality imaging, embryological insights, and an exploration of potential iatrogenic risks. These findings will equip surgeons to improve preoperative planning and ensure intraoperative safety in cholecystectomies and other hepatobiliary, duodenal, or pancreatic procedures. The surgeon's careful attention, precise identification, and confirmation of both cystic arteries before clipping or ligating are crucial. Surgeons should use selective preoperative imaging like CTA in cases such as duodenal or pancreatic procedures where vascular anomalies could complicate surgery, working closely with radiologists to improve detection and surgical planning. By addressing a significant gap in the literature and offering practical guidance for managing this rare anomaly, this study advances the understanding of hepatobiliary anatomy and improves surgical safety and patient outcomes.
